# Long Non-Coding RNA as Potential Biomarker for Prostate Cancer: Is It Making a Difference?

**DOI:** 10.3390/ijerph14030270

**Published:** 2017-03-07

**Authors:** Junli Deng, Jie Tang, Guo Wang, Yuan-Shan Zhu

**Affiliations:** 1 Department of Clinical Pharmacology, Xiangya Hospital, Central South University, Changsha 410008, China; 147311014@csu.edu.cn (J.D.); jietang@csu.edu.cn (J.T.); 2 Institute of Clinical Pharmacology, Central South University, Hunan Key Laboratory of Pharmacogenetics, Changsha 410078, China; 3 Department of Medicine, Weill Cornell Medicine, New York, NY 10065, USA

**Keywords:** prostate cancer, lncRNAs, PCA3, biomarkers

## Abstract

Whole genome transcriptomic analyses have identified numerous long non-coding RNA (lncRNA) transcripts that are increasingly implicated in cancer biology. LncRNAs are found to promote essential cancer cell functions such as proliferation, invasion, and metastasis, with the potential to serve as novel biomarkers of various cancers and to further reveal uncharacterized aspects of tumor biology. However, the biological and molecular mechanisms as well as the clinical applications of lncRNAs in diverse diseases are not completely understood, and remain to be fully explored. LncRNAs may be critical players and regulators in prostate cancer carcinogenesis and progression, and could serve as potential biomarkers for prostate cancer. This review focuses on lncRNA biomarkers that are already available for clinical use and provides an overview of lncRNA biomarkers that are under investigation for clinical development in prostate cancer.

## 1. Introduction

### 1.1. Prostate Cancer Diagnosis and Monitoring

Prostate cancer (PCa) is the most commonly diagnosed noncutaneous malignancy among men in Western countries, and it remains the second leading cause of cancer-associated mortality in that group [1]. According to the Cancer Statistics report, there will be an estimated 161,360 new cases diagnosed and 26,730 deaths in 2017, which represent 19% of all cancer cases and 8% of cancer-related deaths among American men, respectively [2]. Since prostate-specific antigen (PSA) was approved as prostate cancer screening biomolecules by the U.S. Food and Drug Administration (FDA) in 1986, the PCa screening and diagnosis landscape has been revolutionized [3]. Moreover, owing to the utility of a series of complex factors, including PSA in prostate cancer screening and diagnosis in the aging population as well as increased awareness in society, the diagnostic rate of PCa has remarkably increased over the past decades [4].

However, there are inherent limitations to use the PSA test as PCa screening biomolecules [3,5]. Although PSA is expressed specifically in prostate, it is not expressed specifically in prostate cancer, and elevated levels of serum PSA are also found in various noncancerous conditions such as benign prostate hyperplasia, prostatitis, infections, trauma, and urinary retention [5,6,7]. As a result, positive predictive value of PSA screening is only approximately 25%–40% [8]. Due to the false-positive or false-negative results of PSA screening, only about 25% of men who underwent a prostate biopsy based on elevated PSA level (>4.0 ng/mL) were found to have PCa [3]. Moreover, it was shown that the PSA screening is associated with an overdiagnosis and, consequently, an overtreatment of patients with indolent diseases in clinic [9,10,11].

In addition to prostate cancer diagnosis and screening, serum PSA also acts on monitoring prostate cancer progression. The current therapy of prostate cancer is mainly based on androgen receptor activity at almost all the stages of prostate cancer. The standard therapy for hormone-sensitive advanced and metastatic prostate cancer is androgen deprivation therapy (ADT). Although over 80% PCa patients are initially responsive to ADT, a large number of patients suffer from side effects, and after a median of approximately 24 months, advance to castration-resistant prostate cancer (CRPC) [12,13]. Predictive biomarkers would be useful to determine who is most likely to benefit from an individual treatment and to evade treatment failure as much as possible. The serum PSA levels can serve as response indicator for PCa treatment, but it is not suitable or predictive for CRPC development based on serum PSA values.

### 1.2. LncRNAs in Tumor Biology

There are approximately 25,000 protein-coding genes identified by the International Human Genome Sequencing Consortium, which accounts for only about 2% of the human genome [14]. Over 75% of human genome are transcribed and the human transcriptome are mainly classified into five categories [15,16]: (1) protein-coding; (2) long non-coding RNA (lncRNA); (3) read-through (implying a transcript overlapped multiple separate annotated genes); (4) pseudogene; (5) transcript of unknown coding potential (TUCP).

**Figure 1:**
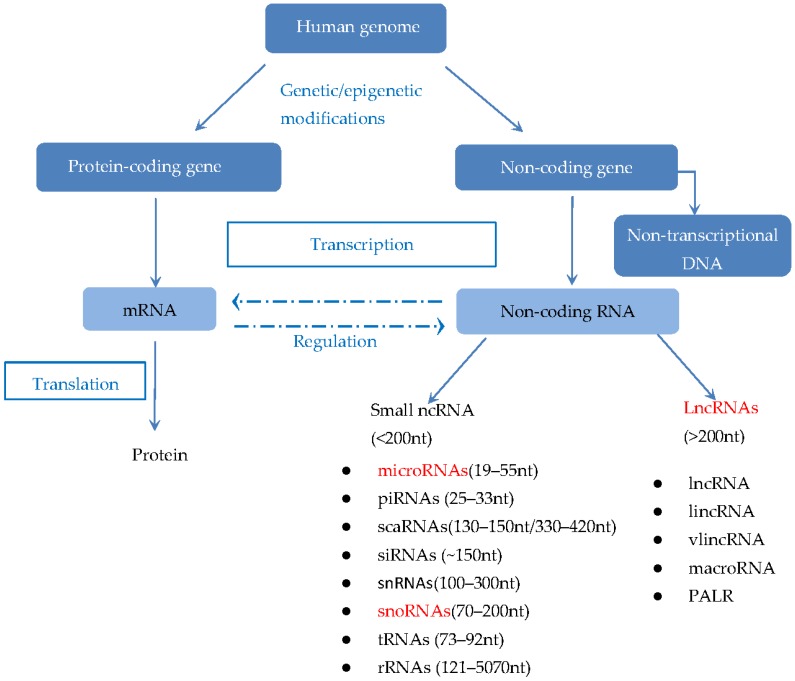
The construction of human genome [17,18,19]. The vast majority of human genome are composed of protein-coding sequences and noncoding sequences. The protein-coding sequences can be transcribed into messenger RNAs (mRNAs) and translated into proteins ultimately, whereas, the nonprotein-coding gene can be transcribed in (functional) noncoding RNAs (ncRNAs) which can be classified in long ncRNAs (lncRNAs) and small ncRNAs based on their size and function. Small ncRNAs comprise microRNAs (miRNAs), piwi-interacting RNAs (piRNAs), ribosomal RNAs (rRNAs), small Cajal body-specific RNAs (scaRNAs), small-interfering RNAs (siRNAs), small nuclear RNAs (snRNAs), small nucleolar RNAs (snoRNAs), and transfer RNAs (tRNAs). Furthermore, tRNAs, rRNAs, snRNAs and snoRNAs belong to the well-characterized housekeeping ncRNAs; miRNAs can also be derived from lncRNAs and snoRNAs (highlighted in red). LncRNAs are mainly classified into lncRNA, lincRNA (long-intergenic non-coding RNA; large intervening non-coding RNA, long-intervening non-coding RNA), vlincRNA (very long intergenic non-coding RNA), macroRNA and promoter-associated long RNA (PALR). In addition, there is a portion of genome that is not transcribed. Adapted from [18].

Non-coding RNAs (ncRNAs) are broadly categorized into small and long ncRNAs based on their differences in size (Figure 1) [17,18,19]. LncRNAs refer to RNAs that have a length greater than 200 nucleotides (nt). Currently, more than 58,000 human lncRNAs have been identified [15]. In comparison to the protein-coding genes, lncRNAs have the characteristics of low sequence conservation across model organisms and low expression levels. Although the vast majority of human genome does not code for any protein and is transcribed into functional ncRNAs eventually, these ncRNAs still play important roles in physiological and pathological processes. It was elucidated that lncRNAs are involved in many biological processes including gene expression regulation, cell cycle regulation, transcription, translation, cellular differentiation, nuclear-cytoplasmic trafficking, as well as chromatin modification [14,20,21,22]. Accumulating studies have demonstrated that lncRNAs may function as oncogenes or tumor suppressors, and play a pivotal role almost at all stages of prostate cancer including initiation, development and progression [23].

### 1.3. Biomarkers

Because of the complexity and heterogeneity of human tumors and the genetic and epigenetic alterations that occur during disease progression, the disease-specific macromolecules are always produced which are effective and predictive to serve as novel biomarkers. It is extremely urgent to explore and discover molecular biomarkers to evaluate disease progression and predict drug response. Potential biomarkers could be specific cells, metabolites, proteins, DNA or epigenetic modification of DNA, and RNA transcripts, including lncRNAs [24]. The ideal and convenient biomarkers should possess several typical and important characteristics. First of all, it should be available for access and collected by means of biopsy or surgical resection. More importantly, it should be detected easily in body fluids such as blood, urine and semen.

The identification of biomarkers has great advantages and priorities in early diagnosis and in discriminating indolent from aggressive PCa. Patients with an indolent disease that has a low risk of progression may benefit from active surveillance and continuous monitoring, while the patients with an aggressive or rapidly progressive PCa will benefit from early diagnosis and treatment with effective therapy. There is an urgent need to develop new diagnostic and predictive biomarkers, which would enable individualized and precise therapeutic management for the prostate cancer patients. Herein, we focus on the promise of lncRNAs as the next-generation diagnostic, prognostic and predictive biomarkers in PCa.

## 2. Commercially-Available Tests for RNAs in Prostate Cancer

Given the high degree of intra-cancer and inter-patient heterogeneity at the molecular level [25], it is an effective approach to profile the expression of multiple genes to establish the molecular processes occurring in the tumor. Herein, we briefly described three commercially available tests in current (and summarized in Table 1) to assess their prognostic ability in stratifying patients at risk of metastasis or biochemical recurrence after primary treatment.

Cuzick et al. [26] tested 126 cell cycle and cellular proliferation genes and found 31 highly correlated genes termed the cell cycle progression (CCP) score. Furthermore, they elucidated that the higher CCP scores are correlated with a higher likelihood of biochemical recurrence or death during a 10-year follow-up. This test, currently available as Prolaris^®^ from Myriad Genetics also has been used in prostate biopsy tissue [27,28]. In a nested case-control study, Erho et al. [29] have analyzed the gene expression in metastatic PCa tissues using microarray analysis, and a Genomic Classifier (GC) composed of 22 genes has been developed. The GC score, currently available as Decipher^®^ Prostate Cancer Classifier from GenomeDx, is positively associated with a greater probability of biochemical recurrence and metastasis even after adjusting for other clinical variables such as Gleason score and seminal vesicle invasion [29,30,31]. In an attempt to identify a gene signature that predicts clinical recurrence, PCa death, and adverse pathology, Klein et al. [32] have adopted a candidate gene approach and analyzed the expression of 732 genes in prostatectomy tissues. As a result, 228 genes were found to be associated with biochemical recurrence, and 81 of the 228 genes were selected for further evaluation in the biopsy cohort. The expression of 17 genes including a gene signature of stromal response, cellular organization, proliferation, and androgen signaling were utilized to calculate the genomic prostate score (GPS). GPS, which is validated and commercially available as OncotypeDX^®^ Prostate Cancer Assay from Genomic Health [33], is also an independent factor that is significantly associated with biochemical recurrence and adverse pathology on prostatectomy [32,34].

## 3. PCA3 and Its Combinations of Multiple Biomarkers

### 3.1. PCA3

With the advancement in transcriptomic analysis, many lncRNAs have been found to be associated with prostate cancer. The search of lncRNA biomarkers in PCa is exemplified by PCA3, a well characterized lncRNA that has been approved by the FDA for clinical decisions about repeat biopsy of prostate cancer [35]. There are two important features making PCA3 an attractive biomarker candidate. Firstly, PCA3 expression is absent in both normal and malignant tissues of non-prostate origin, including genitourinary organs such as kidney, bladder, seminal vesicles, and testis [36,37]. Secondly, PCA3 expression is increased exclusively in patients diagnosed with prostate cancer but not benign prostatic hyperplasia (BPH), prostatic intraepithelial neoplasia (PIN), atypical small acinar proliferation (ASAP), inflammation, or chronic prostatitis [37,38,39]. Bussemakers et al. have shown in 53 out of 56 patients that PCA3 expression in PCa tissues was 10- to 100-fold higher than that in noncancerous tissues collected during radical prostatectomy [36].

The PCA3 lncRNA was first quantified accurately using qRT-PCR analysis of urinary sediments from a cohort of 108 men and recommended as a urinary biomarker for prostate biopsy in patients with a serum PSA value >3 ng/mL [37,40]. Furthermore, Neveu et al. [41] have introduced PCA3 promoter into a new transcriptional amplification system, namely the 3-Step Transcriptional Amplification System (PCA3-3STA), and cloned it to adenovirus, which provides a superior amplification of the PCA3 promoter activity, and has a higher specificity for PCa cells compared to benign primary prostate epithelial cells or non-PCa cells. Moreover, the bioluminescent signals generated by PCA3-3STA have shown to be sufficient to translate to positron emission tomography imaging. Thus, PCA3-3STA, a PCa-specific expression system, has the potential to target primary or metastatic PCa epithelial cells for imaging, vaccines, or gene therapy. Currently, apart from the Prostate Health Index (phi; Beckman Coulter Inc., Brea, CA, USA) assay based on blood, the Progensa^®^ PCA3 Assay from Hologic Gen-Probe (Marlborough, MA, USA) is available for analyzing urinary samples, which provides information for clinicians to decide whether or not to recommend a repeat biopsy [35,42]. A PCA3 score is calculated via Progensa^®^ PCA3 Assay by quantifying PSA and PCA3 transcripts using transcription-mediated amplification in urinary specimens collected post-DRE [36]. A high urinary PCA3 score is correlated with a greater possibility of being diagnosed as prostate cancer on biopsy [43,44,45,46]. Therefore, the Progensa^®^ PCA3 assay, which is far superior to prostate-specific antigen in terms of predictive value and specificity, has significantly improved the outcome of repeat prostate biopsy [43,45,47,48,49,50,51]. Although PCA3 improves the specificity for PCa detection compared to serum PSA, it is not sufficient to use it alone in making a decision for initial biopsy due to its lower sensitivity. Nevertheless, the use of PCA3 assay has improved the clinical diagnosis of PCa even though other new biomarkers are necessary for satisfactory outcome.

### 3.2. Combinations of Multiple Biomarkers

Although lncRNA may be a promising diagnostic biomarker for prostate cancer detection, its accuracy is not necessarily better than that of a biopsy. Considering the discrepancy between a biomarker and the pathological diagnosis, a clinical diagnosis based on biomarkers alone may result in misdiagnosis and unnecessary overtreatment. A combination of multiple biomarker tests could be much better than a single biomarker test for decreasing risks of misdiagnosis and overtreatment. It is anticipated that, in the near future, a combination of multiple biomarker tests will provide a specific, sensitive and accurate detection of prostate cancer.

The PSA test currently used for prostate cancer screening has some inherent shortcomings and limitations [3,5]. Overall, the performance of PSA test is depending on the PSA cutoff values, and the specificity and sensitivity of PSA tests vary from 20% to 40% and 70% to 90%, respectively [24]. The area under curve (AUC) value of PSA, which characterizes a biomarker as a perfect discriminator when it is close to 1, ranges from 0.55 to 0.70 in the receiver operating characteristic (ROC) curve analysis [24], indicating a low and unsatisfactory ability of PSA to identify PCa. Therefore, it is quite urgent to improve the specificity, sensitivity and accuracy of prostate cancer screening through discovering novel biomarkers or integrating present screening tools.

LncRNA PCA3 as an oncogene is the first urinary biomarker test available for PCa diagnosis. A recent study has indicated that PCA3 expression can be considered as a reliable marker for PCa detection based on the fact that a significantly increased PCA3 expression was observed in patients with PCa compared to healthy individuals and patients with benign prostatic hyperplasia (10.64- and 7.17-fold, respectively), while PCA3 expression in both urine and blood samples was not different between control and BPH groups [52]. Tomlins et al. [53] have recently revealed that an incorporation of urinary T2:ERG and PCA3 score has effectively improved the performance of serum PSA for predicting the presence of PCa and high-grade PCa on biopsy (Table 2). Similarly, Rigau et al. [54] have demonstrated that a combination of urinary prostate-specific G-protein coupled receptor (PSGR), a biomarker over-expressed in PCa tissue, and PCA3 test greatly improved the specificity of PCa detection from 15% (PSGR) and 17% (PCA3) to 34% (PSGRvPCA3) at a high sensitivity (95%). Moreover, a combination of fusion gene TMPRSS2:ERG transcripts and PCA3-score in the urine could significantly increase the detection of high risk localized PCa [55]. Incorporation of TMPRSS2:ERG test improves the sensitivity of PCA3 from 68% to 76% in PCa detection, and greatly increases the predictive value of European Randomized Study of Screening for Prostate Cancer (ERSPC) risk calculator for predicting biopsy, Gleason score (*p* < 0.001), and clinical tumor stage (*p* = 0.023) [56]. Actually, a combination of PCA3 and TMPRSS2:ERG test has been shown to reduce the number of unnecessary prostate biopsy and improve the accuracy of PCa diagnosis (Table 3). These data collectively suggest that a combination of current available biomarker tests is a preferred choice to improve the sensitivity, specificity, and accuracy in PCa screening and diagnosis.

## 4. Potential LncRNA Biomarkers under Investigation

Although the study of PCa biomarkers has been primarily focused on the lncRNA PCA3, a variety of other lncRNAs have been identified as being associated to prostate cancer development and progression, which may serve as potential biomarkers (Table 4). These lncRNAs are currently in an early research stage although some of them have been studied in multiple, independently collected cohorts. Nevertheless, the value of these potential biomarkers in PCa screening, diagnosis and prognosis is currently unclear and remains to be evaluated in clinical trials.

### 4.1. LncRNAs and Prostate Cancer Risk Prediction

The difference of genetic variants in populations is one of the important foundations for the risk stratification of diseases. The genetic variants of lncRNAs are strongly related to the risk stratification of patients with PCa.

Previous studies have shown an elevated risk for PCa in West African men compared to European men, which is attributable to the factors of age and genetic susceptibility [85]. Cook et al. [57] performed a genome-wide association study (GWAS) of 474 PCa cases and 458 population-based controls in West African men, and found a strong genetic association with PCa risk located at 10p14 in an intron of an lncRNA (lncRNA RP11-543F8.2), 360 kb centromeric of GATA3 (*p* = 1.29 × 10^−7^) using a multivariable logistic regression analysis. A similar association study of lncRNAs single-nucleotide polymorphisms (SNPs, rs3787016 G > A and rs10773338 G > A) to PCa risk was conducted in an Eastern Chinese population [86]. This study has revealed that the A allele of rs3787016 variant was significantly associated with higher prostate cancer risk (adjusted OR = 1.418 for AA vs. GG). Further analysis indicated that the AG/AA genotype of rs3787016 was significantly associated with patients of younger age, who had smoked, Gleason score ≥7(4 + 3) and highly aggressive cancer [86]. However, all these risks were not present for rs10773338 G > A variant.

Prostate cancer-associated intergenic non-coding RNA transcript 1 (PCAT1), has also been extensively investigated and found to be critical for prostate cancer pathogenesis. This lncRNA resides in chromosome 8q24, a locus that harbors prostate cancer-associated SNPs and exhibits frequent chromosomal amplification [58,59,60]. On the basis of whole-transcriptome analysis, Prensner et al. [61,62] have selected PCAT1 as a top-ranked and upregulated lncRNA in prostate cancer tissues. Guo et al. [63] have recently demonstrated that rs7463708, a prostate cancer risk-associated SNP located 78 kb downstream of the PCAT1 transcription start site (TSS), modulated the activity of PCAT1 enhancer, resulting in an increased PCAT1 expression and consequently, an increased PCa cell proliferation and tumor growth both in in vitro and in vivo system. Prostate cancer gene expression marker 1 (PCGEM1), a lncRNA first reported by Srikantan et al. [64] in 2000 was found to be associated to subjects with a high PCa risk and its expression was significantly higher in African-American men compared to Caucasian-American men (*p* = 0.0002) [65]. Xue et al. have also reported that PCGEM1 polymorphisms, specifically in two tSNPs (tagged single nucleotide polymorphisms) for rs6434568 and rs16834898, are relevant to PCa risk in Chinese men [66]. Taken together, these data suggest that lncRNA SNPs are associated with lncRNA expression and consequently related to PCa susceptibility, which warrants further investigations with a well-selected large population.

### 4.2. LncRNAs as Potential Diagnostic Biomarkers

Neoplastic transcriptomes are far more complicated than what we have previously imagined. Except the dysregulated expression of small non-coding RNAs and protein coding genes, lncRNA dysregulation has been uncovered to be a central contributor to carcinogenesis. Given that lncRNAs are detectable in body fluids like serum, blood, and urine and specifically expressed at various stages of prostate cancer, it would be an unexceptionable choice to take lncRNAs as biomarkers for prostate cancer screening and diagnosis.

Recently, a series of association studies have proven that Metastasis associated lung adenocarcinoma transcript 1 (MALAT1), an lncRNA used to predict metastasis and survival in patients with early-stage non-small cell lung cancer [87], is correlated with prostate cancer development and progression. Ren et al. [67] have reported that the expression of MALAT1 in human prostate cancer tissues and cell lines was closely associated with high PSA levels, Gleason scores, and tumor sizes. Wang et al. [68] have shown that the use of a MALAT1 model, which may serve as an independent predictor of PCa, would prevent unnecessary biopsies in about 30.2%–46.5% of patients with serum PSA levels in the “diagnostic grey zone” (PSA 4–10 ng/mL). Moreover, Ren et al. [69] have found that MALAT-1 derived miniRNA (MD-miniRNA) from plasma may be used as a novel approach to detect human prostate cancer. Compared to non-PCa patients, the plasma MD-miniRNA levels are significantly elevated in PCa patients (*p* < 0.001). At a cut-off of 867.8 copies/mL of plasma MD-miniRNA, the sensitivity and specificity for distinguishing PCa from non-PCa was 58.6% and 84.8%, respectively, and the sensitivity and specificity for distinguishing positive from negative biopsies was 43.5% and 81.6%, respectively. Similarly, Xue et al. [70] have demonstrated that MD-miniRNA had a relatively high diagnostic accuracy with an AUC of 0.86 (95% CI 0.80–0.93) and 0.79 (95% CI 0.70–0.88) to discriminate PCa patients from healthy controls and PCa patients from BPH patients, respectively. Meanwhile, a combination of PSA and MD-miniRNA showed a better diagnostic performance compared to either single biomarker [70]. In sum, these data indicate that MALAT1 is a promising and useful biomarker for prostate cancer detection and warrants further study in clinical trials with a large sample size.

Additionally, other lncRNAs have also been recognized as potential biomarkers for the risk stratification of prostate cancer. LncRNA prostate cancer-associated non-coding RNA transcript 18 (PCAT-18) identified by RNA sequencing is specifically expressed in prostate tissue, and upregulated in prostate cancer compared to other neoplasms and BPH tissues. Like other lncRNAs, PCAT-18 can be detected in plasma, and its expression implicates a progression of prostate cancer. Notably, a silencing of PCAT18 selectively activated caspase 3/7 activity and significantly inhibited cell proliferation, migration, and invasion in PCa cells but not non-neoplastic cells, suggesting that PCAT-18 could function as a potential therapeutic target and biomarker for metastatic prostate cancer [71].

Using RNA-Sequencing, lncRNA FR0348383 has been demonstrated as the most top differentially expressed transcripts among 406 PCa-associated lncRNA transcripts [72]. It was upregulated in 80% (32/40) of patients with prostate cancer and its expression level could significantly differentiate PCa from BPH (*p* = 0.0306) [72]. Zhang et al. [73] have reported that lncRNA FR0348383 level in post-DRE urine could serve as a novel biomarker for PCa detection with an accurate diagnostic value, especially for the subgroup of patients with a PSA value in the grey area. As an independent PCa predictor (*p* < 0.001), the urinary FR0348383 score, defined as the ratio of PSA mRNA and FR0348383 level (PSA mRNA/FR0348383 lncRNA × 1000), shows a much more outstanding performance than PSA and its derivates, including %free PSA and PSA density (AUC: 0.815 vs. 0.562, 0.599, and 0.645, respectively) in the subgroup of patients with a PSA value in the grey area.

Collectively, these data suggest that PCa screening and diagnosis in the future seem to largely rely on the sensitivity, specificity and accuracy of biomarkers, and the potential application of lncRNA biomarkers alone or in combination with other biomarkers might effectively avoid unnecessary prostate biopsy, dramatically increase the specificity of PCa diagnosis, and successfully distinguish PCa patients from indolent diseases.

### 4.3. LncRNA as Potential Prognostic Biomarkers

LncRNA SChLAP1 (second chromosome locus associated with prostate-1; also called LINC00913) is characterized as a tissue-specific biomarker determined by RNA in situ hybridization (ISH) assay [74]. Apart from Gleason score, high expression of SChLAP1 was identified and validated as a significantly prognostic biomarker for metastatic prostate cancer [75]. Moreover, studies over the years have indicated that SChLAP1 expression is increased with prostate cancer progression and the level of SChLAP1 independently predict the poor clinical outcome in patients with localized prostate cancer following radical prostatectomy [74] and patients with lethal prostate cancer [76] through the analysis of hazard ratios (HRs) using multivariable Cox regression. Furthermore, recent studies have indicated that an aberrant upregulation of lncRNA LOC400891 or lnc-MX1-1 in tumor tissues is significantly associated with poorer prognosis and contributes to tumor progression [77,78], suggesting a biological role and clinical significance of lncRNA LOC400891 and lnc-MX1-1 in PCa development and progression. Knockdown of either LOC400891 or lnc-MX1-1 in PCa cells inhibited cell proliferation and invasion [77,78]. Moreover, LOC400891 has been shown to be an independent predictor of biochemical recurrence-free survival in PCa [77]. Meanwhile, over-expression of lnc-MX1-1 is closely relevant to patients’ clinical features including PSA, Gleason score, metastasis, and recurrence free survival [78]. Taken together, these data suggest that both LOC400891 and lnc-MX1-1 may serve as a potential prognostic biomarker and therapeutic target for PCa.

Recently, Maher’s group [79,80] has reported that an aberrant downregulation of prostate cancer associated transcript-14 (PCAT-14) was associated with Gleason score and a greater probability of metastatic progression, overall survival, and prostate cancer-specific mortality across multiple independent datasets and ethnicities. Moreover, studies in in vitro cell cultures demonstrated that a downregulation of PCAT14 increased cell migration while an overexpression reduced cell growth, migration and invasion, suggesting that PCAT14 may possess a significant biological role in PCa tumorigenesis and progression, and may function as a potential biomarker for PCa detection and prognosis.

Except for lncRNAs, exosomes have recently been emerging as a hot spot in biomarker research, and they play an influential role in carcinogenicity and cancer progression. From the perspective of disease detection and prognosis, exosomes exhibit more advantages than current biomarkers. For one thing, cancer cells accumulate and secrete more exosomes than normal cells. For another, exosomes are not only readily regenerated and nonliving, but also can be detected in body fluids such as urine, serum, saliva, and ascites [88]. Furthermore, exosomes package and release various molecular constituents of their cell of origin, such as proteins, lipids, DNA and RNAs, which are involved in cellular communication to modulate target cell activity [89,90]. Owing to the stability and longevity of the urinary exosomal lncRNAs and the lack of reliable biomarkers for PCa detection and classification, it is alternative and ideal to put exosomal lncRNAs as a non-invasive biomarker for diagnosis, prognosis, and tumor characterization. LncRNAs such as MALAT1, HOTAIR, lincRNA-p21, GAS5, TUG1, and CCND1-ncRNA have been detected and shown a differential abundance in secreted exosomes [91]. In a recent study of exosomes conducted with urinary samples from 30 PCa patients and 49 BPH patients, Isin et al. have demonstrated a significant difference in the exosomal lincRNA-p21, but not GAS5 levels between PCa and BPH patients, suggesting that exosomal lncRNA-p21 levels may act as a promising biomarker for the detection of PCa and its distinction from benign disease [81].

## 5. Conclusions

Despite the fact that numerous non-coding transcripts are viewed as inconsequential transcriptional ‘noises’, lncRNAs have recently drawn the widespread attention of many scientists in their search for disease pathogenesis [92,93,94]. Importantly, the study of lncRNAs has gradually constituted one of the most noticeable parts in the field of RNA biology. It is apparent that lncRNAs may possess significant biological functions in prostate physiology and pathophysiology, and play a critical role in PCa development, progression, diagnosis, prognosis, and management.

LncRNAs have been used in the clinic as a biomarker for PCa diagnosis, and a large number of lncRNAs in the research phase may become useful biomarkers in the near future. Based on the information to date, a combination of multiple biomarkers is a superior choice in PCa screening, diagnosis, prognosis, and therapeutic monitoring. Given the roles of lncRNAs in prostate cancer, it will be meaningful to develop lncRNAs as useful biomarkers through well-designed clinical trials, and create new agents that target specific lncRNA signaling networks related to PCa development and progression. It is extremely likely to incorporate lncRNA analysis to improve the specificity and sensitivity of existing biomarkers. The advancement of new technology such as quantitative real-time PCR, RNA-seq and RNA-FISH will undoubtedly facilitate the translation of lncRNA analysis to the bedside, which will certainly make a difference in PCa screening, diagnosis, and management.

## Figures and Tables

**Table 1:** Commercially available mRNA test for prostate cancer.

References	Name (Vendor)	Number of Genes	Discovery Approach
[26]	Prolaris^®^ (Myriad Genetics)	31 CCP test genes	Candidate gene approach, 126 cell cycle and cellular proliferation genes tested in prostatectomy tissues
[29]	Decipher^®^ Prostate Cancer Classifier (GenomeDx)	22 GC test genes	Whole transcriptome analysis Affymetrix GeneChip^®^ Human Exon 1.0 ST Array used to assess prostatectomy tissues
[32]	OncotypeDX^®^ Prostate Cancer Assay (Genomic Health)	12 GPS test genes	Candidate gene approach, 727 genes tested in prostatectomy tissues Refined panel of 81 genes tested in biopsies

CCP: Cell Cycle Progression; GC: Genomic Classifier; GPS: Genomic Prostate Score.

**Table 2:** Performance of serum prostate-specific antigen-based models for predicting cancer and high-grade cancer on biopsy (Adapted from [53]).

Model	Prediction	*n*	AUC	*p* Value vs. PSA	*p* Value vs. T2:ERG or PCA3
PSA	Cancer	1225	0.585	NA	NA
PSA plus T2:ERG			0.693	<0.001	NA
PSA plus PCA3			0.726	<0.001	<0.05
PSA plus T2:ERG plus PCA3 (MiPS)			0.751	<0.001	<0.001, <0.001
PSA	HG cancer	1225	0.651	NA	NA
PSA plus T2:ERG			0.729	<0.001	NA
PSA plus PCA3			0.747	<0.001	NS
PSA plus T2:ERG plus PCA3 (MiPShg)			0.772	<0.001	<0.01, <0.001

AUC: area under the curve; HG: high grade; MiPS: Mi-Prostate Score; MiPShg: Mi-Prostate Score, high grade; NA: not available; NS: not significant; PSA: prostate-specific antigen; T2:ERG: TMPRSS2:ERG; PCA3: prostate cancer antigen 3.

**Table 3:** Clinical implications of PCA3 plus TMPRSS2-ERG assays (adapted from [56]).

Model	Prostate Biopsies Avoided (*n* = 443) *n* (%)	Prostate Cancers Missed (*n* = 196) *n* (%)	Prostate Cancers Gleason ≥ 7 Missed (*n* = 115) *n* (%)
PCA3 score ≥ 25	166 (37)	37 (19)	20 (17)
PCA3 score ≥ 35	211 (48)	62 (32)	36 (31)
TMPRSS2-ERG ≥ 10	382 (86)	150 (77)	75 (65)
PCA3-25 plus TMPRSS2-ERG	153 (35)	26 (13)	11 (10)
PCA3-35 plus TMPRSS2-ERG	195 (44)	48 (24)	24 (21)

TMPRSS2-ERG: v-ets erythroblastosis virus E26 oncogene homolog; PCA3 score: [copies PCA3 mRNA]/[copies PSA mRNA] × 1000; TMPRSS2-ERG positive: ≥ 10 copies TMPRSS2-ERG mRNA; PCA3-25 plus TMPRSS2-ERG: TMPRSS2-ERG positive and/or PCA3 ≥ 25; PCA3-35 plus TMPRSS2-ERG: TMPRSS2-ERG positive and/or PCA3 ≥ 35.

**Table 4:** Potential lncRNA biomarkers under investigation in prostate cancer.

References	lncRNA	Alteration in Prostate Cancer	Location	Clinical Association	Description
[57]	lncRNA RP11-543F8.2	Unknown	Unknown	Risk prediction	A set of promising susceptibility loci
[58,59,60,61,62,63]	PCAT1	Upregulation	Tissues, plasma	Risk prediction	Promoting prostate cancer cell proliferation and tumor growth
[64,65,66]	PCGEM1	Upregulation	Tissues	Risk prediction	Polymorphisms were associated with an increased risk of prostate cancer
[67,68]	MALAT1	Upregulation	Tissues, urinary	Diagnosis	Preventing unnecessary biopsies
[69,70]	MALAT-1 derived miniRNA (MD-miniRNA)	Upregulation	Plasma	Diagnosis	Higher sensitivity, specificity, and accuracy
[71]	PCAT-18	Upregulation	Tissues, plasma	Diagnosis	A potential therapeutic target and biomarker for metastatic prostate cancer
[72,73]	lncRNA FR0348383	Upregulation	Tissues	Diagnosis	More outstanding performance than PSA
[74,75,76]	SChLAP1	Upregulation	Tissues	Prognosis	Independently predicting the poor clinical outcomes
[77]	lncRNA LOC400891	Upregulation	Tissues	Prognosis	As an independent predictor for biochemical recurrence-free survival of PCa
[78]	lnc-MX1-1	Upregulation	Tissues	Prognosis	Relevant to patients‘ clinical features
[79,80]	PCAT14	Upregulation	Tissues	Prognosis	Highly expressed in low grade disease and loss of PCAT14 predicts for disease aggressiveness and recurrence.
[81]	lincRNA-p21	Upregulation	Exosomes	Prognosis	A promising prognostic biomarker for the detection and stratification of PCa
[82]	CCAT2	Upregulation	Tissues	Prognosis	High CCAT2 expression level had poorer overall survival and progression-free survival
[83]	HCG11	Downregulation	Tissues	Prognosis	Downregulation of HCG11 expression in tissues was associated with poor survival of PCa patients.
[84]	ATB	Upregulation	Tissues	Prognosis	High lncRNA-ATB expression may be an independent prognostic factor for biochemical recurrence (BCR)-free survival in prostate cancer patients.
